# A combined subsegmentectomy of right S9 + 10b with single direction thoracoscopic surgery

**DOI:** 10.1186/s13019-024-02676-0

**Published:** 2024-04-13

**Authors:** Cheng Shen, Chengwu Liu

**Affiliations:** Department of Thoracic Surgery, West-China Hospital, Sichuan University, Chengdu, 610041 China

**Keywords:** Thoracic surgery, VATS, Combined subsegmentectomy, Ground glass opacity, Single-direction thoracoscopic surgery

## Abstract

Segmentectomy is widely used to treat pulmonary nodules and more functional lungs can be preserved in patients. For pulmonary nodules deep near the intersegmental border, only one single segmentectomy may not achieve adequate surgical margins, and combined subsegmental resection becomes the most suitable treatment option. Thoracoscopic combined anatomical resections involving both of right S9 and S10b are one of the most challenging cases, especially in the right chest. We previously reported a case of combined subsegmental resection of the left complex basal segment (LS9b + 10b). To our knowledge, there has been no report of combined subsegmental resection of the right S9 and S10b. Here, we aim to introduce a different technique named as “open-gate”, which means that the intersegmental border between S7 and S10 was cut open along the intersegmental septa, to deal with complex combined basal subsegmental resections.

**Supplementary Information:**

The online version contains supplementary material available at 10.1186/s13019-024-02676-0.

## Background

Segmentectomy is widely used to treat pulmonary nodules and more functional lungs can be preserved in patients. For pulmonary nodules deep near the intersegmental border, only one single segmentectomy may not achieve adequate surgical margins, and combined subsegmental resection becomes the most suitable treatment option. Thoracoscopic combined anatomical resections involving both of right S9 and S10b are one of the most challenging cases, especially in the right chest [1, 2]. We previously reported a case of combined subsegmental resection of the left complex basal segment (LS9b + 10b)^3^. Here, we aim to share a case of combined subsegmental resection to introduce techniques to address complex combined basal segmental resections.

## Case report

A 50-year-old woman was admitted to our hospital for assessment of a ground glass opacity (GGO) nodule (8*8 mm) in the right lower lobe revealed by the high-resolution computed tomography (HRCT). No radiologic changes had occurred at the 8 month follow-up. Informed written consent and institutional review board approval were both obtained (No. 2021 − 879; July 25, 2022) for the surgery and the publication of the study data.

**Fig. 1:**
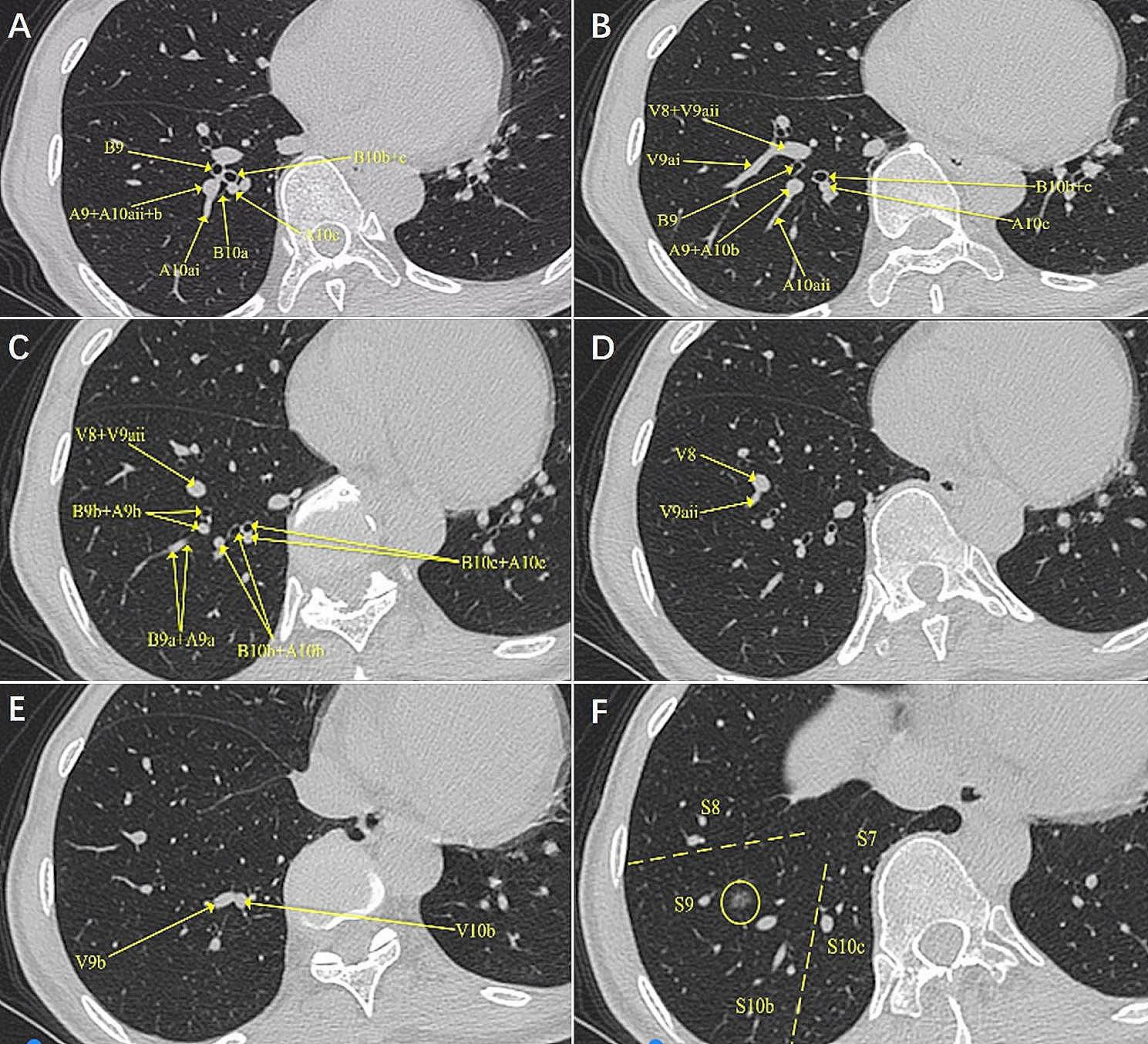
Identifying the subsegmental structures on preoperative HRCT. **A**, Identifying B9 and B10. **B**, Identifying V9ai and V9aii. **C**, Identifying A9a, A9b, and A10b + c. **D**, Identifying V8 and V9aii. **E**, Identifying V9b and V10b. **F**, Showing the extent the right S9 + 10 by bcombined subsegmentectomy

**Fig. 2:**
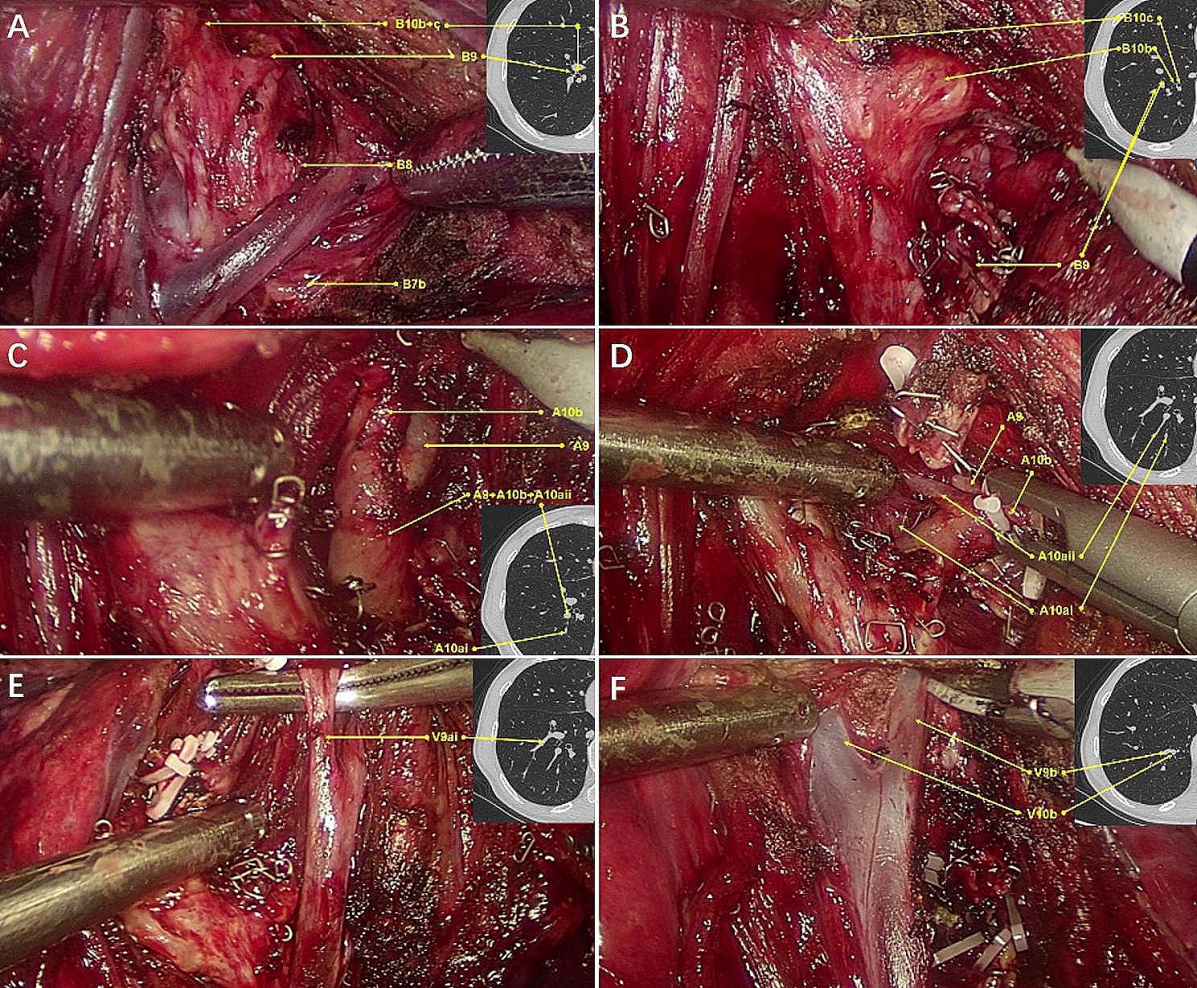
The single-direction combined basal subsegmentectomy of right S9 + 10b through the inferior pulmonary ligament approach. **A**, Identifying the basal segmental bronchus and B9 and B10b. **B**, Disconnecting the B9 bronchus and identifying B10b and B10c. **C**, Dissecting the inferior pulmonary artery and identifying the intersubsegmental vein of A9 and A10b. **D**, Dissecting and managing A9 and A10b. **E**, Identifying and managing V9ai. **F**, Identifying and managing V9b and V10b

Then, the surgical plan was made mainly based on the preoperative HRCT (Fig. 1, A-E). A combined subsegmentectomy (S9 + S10b) was planned (Fig. 1, F). The details of the procedures are introduced in Video. The surgery was initiated by dissecting the inferior pulmonary ligament and then the basal segmental vein and its branches. Then, the intersegmental border between S7 and S10 was cut open along the intersegmental septa. Only after that can we approach the deep segmental and subsegmental hili of S9 and S10b. This was called the open-door process. After that, the procedure proceeded in a single direction manner from the caudal side to the cranial side and from the superficial to deep structures. The target subsegmental structures of B9 and B10b (Fig. 2, A and B), A9 and A10b (Fig. 2, C and D), V9a and V9b (Fig. 2, E and F), and V10b (Fig. 2, F) were dissected and transected in order of appearance. After that, the intersubsegmental demarcation line was identified by the modified inflation–deflation method and the intersubsegmental planes were managed using stapler-based tailoring method. Finally, the thoracoscopic combined basal subsegmentectomy of RS9 and 10b was completed. Systemic mediastinal lymph node dissection was performed, with stations 2, 4, 7, 8, 9, 10, 11 and 13 lymph nodes removed.

After complete resection of the lesion, tissue of the nodule was taken out from the tumor for quick frozen pathology. Intraoperative frozen section pathologic examination documented an adenocarcinoma. The operation time was 120 min, and the intraoperative blood loss was 15 ml. The postoperative course was uneventful, and the patient was discharged on postoperative day 3 after the operation with no complication. The final pathologic examination documented a minimally invasive adenocarcinoma with negative surgical margin, pT1aN0M0. She had been followed up for one month without evidence of recurrence.

## Discussion

For pulmonary nodule diagnosed as GGO on imaging, wedge resection and anatomic segmentectomy are both options if adequate margins can be obtained during surgery, and they can also provide patients with good prognosis and respiratory function status [3–5]. Occasionally, lung lesion may appear at the intersegmental boundary between two segments. In this case, resection of either segment is not sufficient to provide a negative resection margin. In our patient, for example, the lesion was a subcentimeter pure GGO next to the subsegmental border between S9 and S10b. Based on imaging features, early-stage lung adenocarcinoma is highly suspected, and after comprehensive evaluation, it is recommended that the patient needs surgery. After careful analysis of HRCT, we found that combined subsegmentectomy of S9 and S10b could provide safe margins and help to preserve subsegments of S10a and S10c. Finally, our surgical protocol was a combined subsegmentectomy for this patient.

In minimally invasive surgery, basal segmental resection of the lung is particularly challenging because lung structures are located deep within the lung parenchyma, anatomical variations may exist in different patients, and delineation of the intersegmental plane is difficult [6, 7]. As in our case, S9 and S10b are sub-segments inside the right basal segment. In previous studies, we have described in detail the technical procedure for single direction thoracoscopic basal segmentectomies using the transinferior ligament approach and the stem-branch method in detail [8]. However, in our case, the surgical procedure was much more complicated because two segments and sub-segments needed to be treated simultaneously and the target structure was located much deeper in right lower lobe. The intersegmental border between S7 and S10 was cut open along the intersegmental septa. Only after that we can approach the deep segmental and subsegmental hili of S9 and S10b. This was called the open-door process. We dissected along the stems of the basal bronchi and vasculature, tracking their branches and identifying them using a stem-branch approach based on their positional relationships obtained in preoperative planning. Finally, we successfully performed this complex combined basal subsegmentectomy. The advantage of the open-door method is that in the face of the intricate surgical field, it can be exposed to the operator as much as possible, so that the targeted lung segment resection can be completed more accurately and other lung segments that should be preserved can be avoided by mistake. The limitation of this technique is that it requires surgeons to be very familiar with the anatomical structure of the lungs, which can be challenging for beginners.

In conclusion, thoracoscopic combined basal subsegmentectomy of RS9 + 10b can be performed through the inferior pulmonary ligament approach by using the method of stem-branch in a single-direction manner.

### Electronic supplementary material

Below is the link to the electronic supplementary material.

Supplementary Material 1

## Data Availability

All data for this study are publicly available and are ready for the public from database of hospital.
